# Phylogenetic identification of marine bacteria isolated from deep-sea sediments of the eastern South Atlantic Ocean

**DOI:** 10.1186/2193-1801-2-127

**Published:** 2013-03-22

**Authors:** Marcus Adonai Castro da Silva, Angélica Cavalett, Ananda Spinner, Daniele Cristina Rosa, Regina Beltrame Jasper, Maria Carolina Quecine, Maria Letícia Bonatelli, Aline Pizzirani-Kleiner, Gertrudes Corção, André Oliveira de Souza Lima

**Affiliations:** 1 Centro de Ciências Tecnológicas da Terra e do Mar, Universidade do Vale do Itajaí (UNIVALI), Rua Uruguai, 458, Itajaí, SC, CEP 88302202 Brazil; 2 Departamento de Microbiologia, Imunologia e Parasitologia, Instituto de Ciências Básicas da Saúde, Universidade Federal do Rio Grande do Sul (UFRGS), Rua Sarmento Leite 500, Cidade Baixa, Porto Alegre-RS, CEP 90050-170 Brazil; 3 Departamento de Genética, Universidade de São Paulo, Escola Superior de Agricultura “Luiz de Queiroz”, Av. Padua Dias, 11, Piracicaba, SP, CEP 13418-260 Brasil

**Keywords:** Deep-sea sediments, South Atlantic Ocean, Phylogenetic identification, Cultivable bacteria, Halomonas

## Abstract

The deep-sea environments of the South Atlantic Ocean are less studied in comparison to the North Atlantic and Pacific Oceans. With the aim of identifying the deep-sea bacteria in this less known ocean, 70 strains were isolated from eight sediment samples (depth range between 1905 to 5560 m) collected in the eastern part of the South Atlantic, from the equatorial region to the Cape Abyssal Plain, using three different culture media. The strains were classified into three phylogenetic groups, Gammaproteobacteria, Firmicutes and Actinobacteria, by the analysis of 16s rRNA gene sequences. Gammaproteobacteria and Firmicutes were the most frequently identified groups, with *Halomonas* the most frequent genus among the strains. Microorganisms belonging to Firmicutes were the only ones observed in all samples. Sixteen of the 41 identified operational taxonomic units probably represent new species. The presence of potentially new species reinforces the need for new studies in the deep-sea environments of the South Atlantic.

**Electronic supplementary material:**

The online version of this article (doi:10.1186/2193-1801-2-127) contains supplementary material, which is available to authorized users.

## Background

Deep-sea environments are the largest continuous ecosystems on our planet. Except for specific places considered diversity hot spots, such as hydrothermal vents and cold seeps, other deep-sea environments including the abyssal plains are in most cases scarcely studied (Jorgensen and Boetius 2007).

The abyssal plains are located between 3000 and 5000 meters deep, and cover 54% of the planet’s surface. A big part of this region is covered with biogenic sediments, although hard substrata such as manganese nodules may be found in some places. Characteristically, the biota of abyssal plains is limited by food, since this environment is devoid of primary production and depends on the input of organic matter from surface waters. Because of its extent and difficulty of access, the abyssal plains are among the least understood ecosystems of our planet, despite the great diversity that these environments may harbor (Smith et al. 2008).

Most of the research on microbial diversity conducted on sediment samples from abyssal plains are based on cultivation-independent methods, which are considered more appropriate, since they allow the detection of the non-cultivable microorganism that are generally constitute the largest fraction of the total microbial community (Polymenakou et al. 2009; Schauer et al. 2010). However, cultivation-based studies may provide information on the physiological characteristics of the organisms living in the sediments, and allow the description of new species and the prospecting for microorganisms of biotechnological interest ( Zengler et al. 2002; Pettit 2011). Gärtner et al. (2011) isolated and identified 107 bacteria from deep sediments of the Mediterranean Sea, and most of the strains belonged to two phylogenetic groups, Firmicutes and Actinobacteria. The most frequently identified genus was *Bacillus*, and 13 possibly new species were also detected. Most of the isolated strains were capable of growing under conditions similar to those prevailing at the source of the samples, relating to temperature and nutrient concentrations.

Aiming to find microorganisms with potential for lipase production, Zeng et al. (2004) isolated lipolytic microorganisms from samples of deep marine sediments from the Eastern Pacific Ocean. Most of the bacteria isolated belonged to the genera *Halomonas*, *Psychrobacter*, *Stenotrophomonas*, *Pseudomonas* and *Pseudalteromonas* of Gammaproteobacteria. Some of the strains produced several types of hydrolytic enzymes, which demonstrated the potential of microorganisms from deep-sea sediments.

The Atlantic Ocean is younger and less tectonically active than the Pacific Ocean. The mid-oceanic ridge is the most important feature of the Atlantic Ocean, and separates it in two halves. Each half of the South Atlantic is further separated in other basins by smaller ridges and other features. On the eastern side, which is the focus of this work, two features are important, the Romanche Trench, in the equatorial region, and the Walvis Ridge, located further south. Between these features is the Angola Abyssal Plain and, south of the Walvis Ridge, the Cape Abyssal Plain (Levin and Gooday 2003).

The Walvis Ridge is a discontinuous submarine feature that intercepts the African continental margin at a latitude of 20°S, extending to the mid-ocean ridge at 37°S. The Walvis Ridge has a characteristic oceanic crust composition, with twice the normal thickness, about 12 to 15 km (Hekinian 1974; Salters and Sachi-Kocher 2010). The origin of the Walvis Ridge is still in debate, but it seems to be associated with the interaction of the Tristão da Cunha hot spot and the movement of the overlying tectonic plate. It is believed that this aseismic ridge represents the beginning of a hot spot trail with southward movement, formed from a fissure of the oceanic crust during the movement of the American and African tectonic plates (Hekinian 1974; Elliott et al. 2009). The Angola and Cape Abyssal Plains are separated by the Walvis Ridge, which functions as a barrier that limits the water flow between these two oceanic basins. The North Atlantic Deep Water, which originates in the Arctic, dominates the Angola Basin. The sediments in this region are typical of deep-sea environments, comprising mainly siliceous and calcareous microfossils. The Low Circumpolar Deep Water, which originates in the Antarctic continent, dominates the Cape Basin (Jansen et al. 1984; Schauer et al. 2010). All these features may influence the distribution of microbial species living in the sea floor and at the deep-sea ecosystems in general.

The deep-sea environments are less studied concerning their microbial diversity, in comparison to other ecosystems, and this applies to the South Atlantic. Recently, Schauer et al. (2010) studied microbial diversity and its biogeography in some basins of the Eastern South Atlantic. These investigators reported the dominance of Proteobacteria, Gammaproteobacteria in particular, in the Guinea, Cape and Angola Basins. Other phylogenetic groups were also identified. The communities of cultivable bacteria were not studied.

In short, there is a paucity of studies on cultivable bacteria from the abyssal plains, despite the importance to biotechnology (Arahal and Ventosa 2006; Simon-Colin et al. 2008; Pettit 2011), for the description of new species and to understand physiological aspects that are relevant to the survival of deep-sea microorganisms. Therefore, the aim of this study was the isolation of microorganisms from sediment samples collected on the abyssal plains of the Eastern South Atlantic Ocean and their phylogenetic identification.

## Results

Colony counts of the samples, as determined in MA, varied between 23 and 1.41 × 10^6^ CFU/g of sediment. The number of isolated strains per sample varied between 4 (sample 5) and 15 (sample 1) (Table 1).No tendencies were observed in these counts, in relation to the depth and location of the sampling stations. Seventy microorganisms were isolated from the three culture media employed. Thirty-five were isolated from MA plates, 18 from MA+Tween 40 plates and 17 MA+CMC plates.

**Table 1:** **Colony counts (MA plates) and number of strains obtained from the three culture media employed**

Sample	Number of isolates	CFU/g
1	15	1410000
2	5	1172500
3	10	1533
4	7	467
5	4	43111
6	10	193333
7	6	23
8	13	27

**Figure 1:**
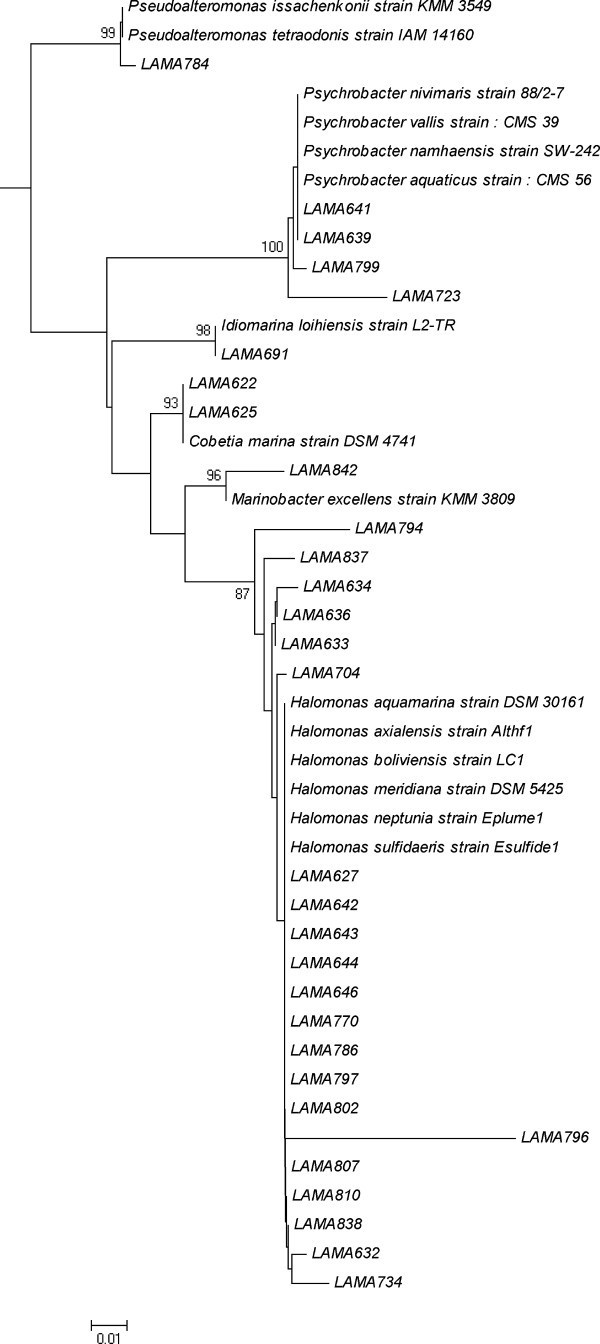
**Neighbor-joining tree showing the phylogenetic relationships of 16S rRNA gene sequences of Gammaproteobacteria strains.** Legend: Bootstrap support values over 70% are shown. The scale bar indicates evolutionary distance.

**Figure 2:**
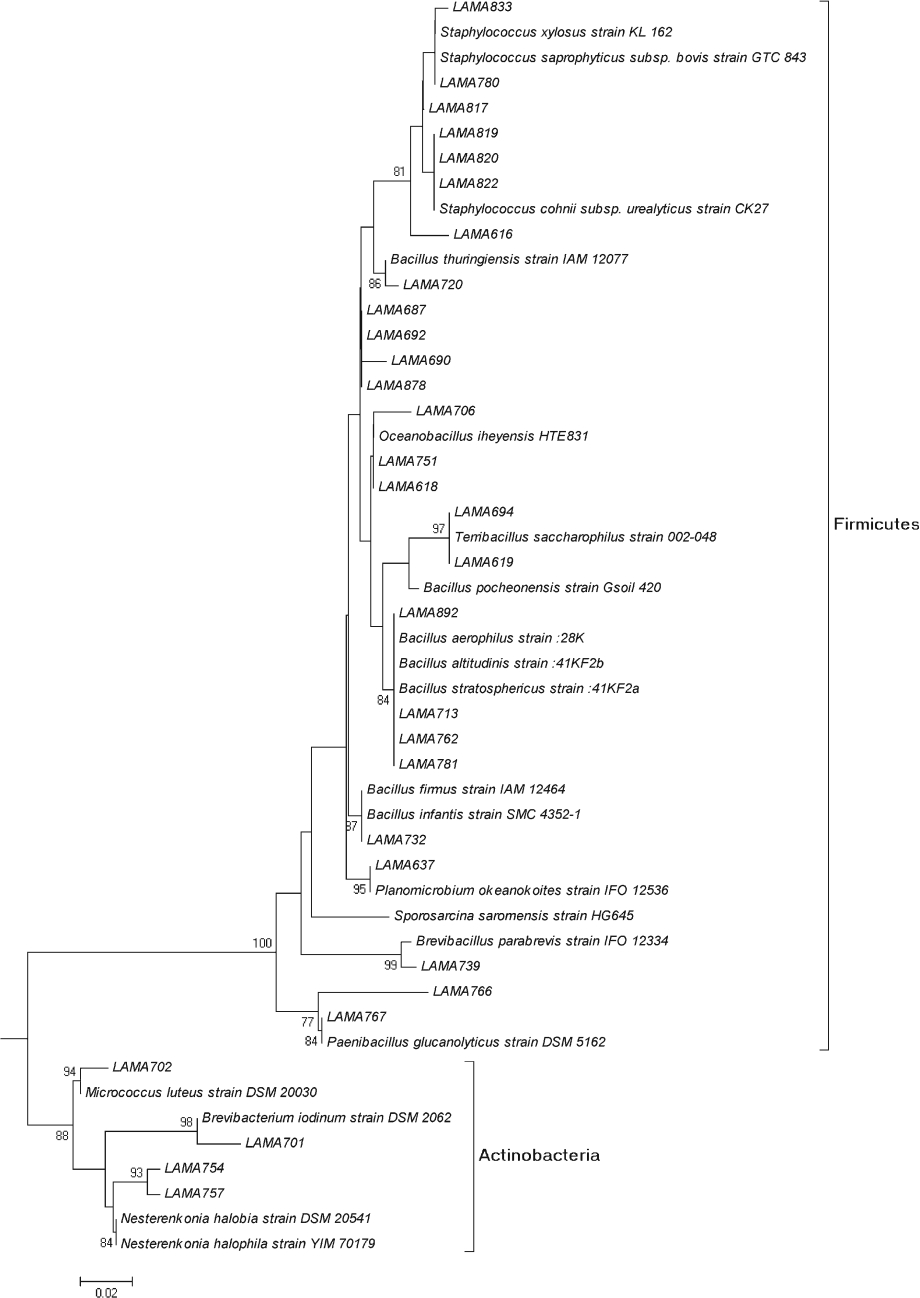
**Neighbor-joining tree showing the phylogenetic relationships of 16S rRNA gene sequences of Firmicutes/Actinobacteria strains.** Legend: Bootstrap support values over 70% are shown. The scale bar indicates evolutionary distance.

According to the partial sequencing of the 16S rRNA gene, the 70 isolated microorganisms were identified as belonging to three phylogenetic groups, Gammaproteobacteria, Firmicutes (Gram-positives with low G+C percentage) and Actinobacteria (Gram-positives with high G+C percentage) (Figures 1 and 2). Most of the strains belonged to Gammaproteobacteria (39 strains) and Firmicutes (27 strains). Only four strains belonged to Actinobacteria. The only phylogenetic group detected in all samples was Firmicutes. Microorganisms from the Gammaproteobacteria class were not detected in sample 5 (sample from 70 cm below the surface of the sediment), in which only Firmicutes were detected. Actinobacteria were detected in samples 1, 3 and 7.

Forty-one different OTUs were defined among the 70 isolated strains Additional file 1. Sixteen OTUs may represent new species, since they showed less than 97% similarity to sequences in the database / published sequences, each of them containing only one strain. Nineteen OTUs were identified in the Gammaproteobacteria class, with 10 belonging to the genus *Halomonas*. Six OTUs of *Halomonas* may represent new species. Three OTUs were attributed to *Psychrobacter*, with two representing new species, and three OTUs were related to *Idiomarina*, with two possibly new species.

Eighteen OTUs were identified in Firmicutes, six belonging to *Bacillus* and four to *Staphylococcus*. Two OTUs were related to the genera *Paenibacillus* and two others to *Oceanobacillus*. Five OTUs of Firmicutes may represent new species, belonging to the genera *Bacillus*, *Oceanobacillus*, *Paenibacillus* and *Staphylococcus*. Each strain of Actinobacteria represented a different OTU, with one possibly being a new species. Two OTUs of Actinobacteria were related to the genus *Nesterenkonia*.

The number of OTUs per sample reflected in an approximate way the number of strains isolated. A small number of OTUs were identified in samples with a small number of strains. For instance, two OTUs were obtained from sample 5, from which only four strains were obtained. This was the only sample collected below the surface of the sediment (70 cm below the surface). A higher number of strains (n = 15) were isolated from sample 1, which also showed the highest number of OTUs. This sample was collected at the Cape Abyssal Plain, at the highest latitude sampled in this study.

Only nine out the 41 identified OTUs were detected in more than one sample (Table 2). The OTUs 7 (related to *Halomonas boliviensis* strain LC1) and 23 (related to *Bacillus aerophilus* strain :28K/*B. altitudinis* strain :41KF2b/*B. stratosphericus* strain :41KF2a) were observed in four samples. The highest numbers of unique OTUs were observed in samples 1 and 6 (n = 8 and 6). No unique OTUs were observed in sample 5.

**Table 2:** **Numbers of each OTU identified in more than one sample analyzed**

OTUs (number and description)	Sample							
	1	2	3	4	5	6	7	8
1, *Cobetia marina* strain DSM 4741						1	1	
7, *Halomonas boliviensis* strain LC1			4	1		1		4
9, *Halomonas sulfidaeris* strain Esulfide1			1			1		1
13, *Idiomarina loihiensis* strain L2-TR	5					1		
21, *Bacillus firmus* strain IAM 12464				1				4
23, *Bacillus pocheonensis* strain Gsoil 420	1			1	1			1
27, *Oceanobacillus iheyensis* HTE831	1			1				
31, *Planomicrobium okeanokoites* strain IFO 12536		1					1	
37, *Terribacillus saccharophilus* strain 002-048					3			1

**Figure 3:**
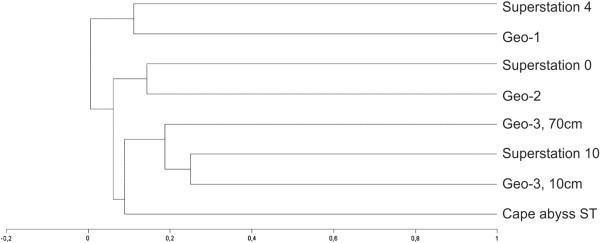
**Dendogram showing the similarity of the samples, accordingly to the presence/absence of the established OTUs.** Legend: Similarities were calculated using the Jaccard coefficient and UPGMA linkage. Group 1 compromise samples 2 and 7, group 2, samples 3 and 6, and group 3 samples 1, 4, 5 and 8.

By cluster analysis, the samples could be separated into three groups (Figure 3). The first group comprised samples 2 (Angola Abyssal Basin) and 7 (South Equatorial Region), both originating from further north. The second group included samples 3 (Cape Abyssal Plain) and 6 (Angola Abyssal Basin). Finally, the third group comprised samples 1, 4, 5 (all originating from the Cape Abyssal Plain) and 8 (Walvis Ridge Region). Most of the samples collected south of the Walvis Ridge (except sample 3), were in the third group of the cluster. This group also included samples 4 and 5, collected at the same station, but at different depths of the sediment.

## Discussion

Microorganisms belonging to Gammaproteobacteria were dominant in the samples studied. This is not unexpected considering that bacteria from this phylogenetic group are among the most known and readily cultivable microorganisms from the marine environment (Fuhrman and Hagström 2008), and this result is in agreement with reports from other studies on diverse marine environments (; Kobayashi et al. 2008; Ettoumia et al. 2011; Finnegan et al. 2011). In the culture–independent study of Schauer et al. (2010) conducted at the Angola, Guinea and Cape Basins, Gammaproteobacteria was also the dominant group, although most of the phylotypes identified belonged to not yet cultivated species.

The only sample in which we could not detect Gammaproteobacteria was sample 5, collected at 70 cm below the surface of the sediment. This observation may be related to the fact that most of the marine bacteria from this phylogenetic group are aerobic or facultative anaerobic (Fuhrman and Hagström 2008), and their occurrence is favored on the surface of the sediment, where oxygen is more abundant. We did not measure the oxygen concentration in the samples studied, but it is reasonable to presume that this element was absent in sample 5, considering the distribution of dissolved oxygen in marine sediments (Hensen et al. 2006).

Firmicutes was the second group most frequently isolated from the samples studied. This group and Actinobacteria comprise the Gram-positive bacteria. Recent studies have reported a greater presence of Gram-positive bacteria in marine sediments (Zhuang et al. 2003; Gontang et al. 2007; Sass et al. 2008), and in several cases these microorganisms are dominant among the cultivable bacteria (Toffin et al. 2004; Gärtner et al. 2011; Velmurugana et al. 2011). Most of the 27 strains identified in Firmicutes were related to endospore-forming bacteria (EFB, 19 strains), mainly *Bacillus*. The ability to produce endospores and the great metabolic and physiological diversity are two characteristics of the EFB that allow their distribution in all environments of our planet (Priest 1993), and this may explain the detection of these bacteria in all samples analyzed in our study. Some of the OTUs (25 identified as *Bacillus thuringiensis*, for instance) were identified as non-marine species of the EFB, and it is probable that these strains were latent in the sediments studied. However, other OTUs (27 and 28) were identified in the genus *Oceanobacillus*, which was originally described from deep-sea sediments (Lu et al. 2001). Therefore, some of the strains of EFB are probably true members of the communities of cultivable bacteria of deep-sea sediments.

The genus most frequently identified among the isolated strains was *Halomonas* (Gammaproteobacteria). This result was expected considering that this genus is widespread in the marine environment, and its cultivation has been reported in several types of samples collected from deep-sea ecosystems, including sediments from hydrothermal vents, from abyssal plains and sub-surface, invertebrates and water, sometimes as dominant microorganisms (Xu et al. 2005; Kaye and Baross 2000; Kaye et al. 2004; Simon-Colin et al. 2008; Kaye et al. 2011). The widespread occurrence of *Halomonas* has been associated with its great physiological and metabolic versatility, which was demonstrated by their capability to assimilate a great variety of carbon sources, and by the growth of these bacteria in a wide range of salinities, temperatures and pressures (Okamoto et al. 2004; Kaye and Baross 2004). These characteristics may not only explain the dominance of this genus, but also its occurrence in the samples studied. The metabolic versatility of *Halomonas* has also been associated with a great biotechnological potential, for production of enzymes and polyhydroxyalcanoates, for instance (Arahal and Ventosa 2006; Simon-Colin et al. 2008).

*Psychrobacter* and *Idiomarina* were the two other genera of Gammaproteobacteria more frequently identified among the strains. The detection of these genera in our samples is consistent with other studies. The genus *Idiomarina*, for instance, was originally described for strains isolated from deep-sea waters (Ivanova et al. 2000 , and lately, another species has been described from a submarine volcano nearby the Hawaii Islands, and was named *Idiomarina loihiensis* (Donachie et al. 2003) . Other investigators also reported the occurrence of *Psychrobacter* in deep-sea environments (Maruyama et al. 2000; Xu et al. 2005). Therefore, bacteria from these two genera may be considered native members of deep-sea communities.

Fifteen percent of the strains were isolated from the MA plates, without supplementation with CMC or Tween 40. The incorporation of CMC or Tween 40 in the culture media could have favored the growth of few organisms especially adapted to the utilization of these carbon sources or adapted to a higher concentration of carbon. These organisms may have inhibited the growth of other species, a phenomenon reported especially in Gammaproteobacteria (Gärtner et al. 2011), the dominant group among our strains. This hypothesis may explain the lower number of strains isolated from MA+CMC and MA+Tween40 plates.

The highest numbers of OTUs were detected in samples 1, collected at the Cape Abyssal Plain, and 6, collected at the Angola Abyssal Plain. Both samples also had the highest number of unique OTUs. These observations may be associated with local enrichments of the sediments with organic matter and nutrients in general, which are associated with the import of primary production from the overlying waters (Orcutt et al. 2011) and this region is characterized by a higher annual primary production (Gregg et al. 2003) in comparison to more tropical or central areas, resulting in a greater exportation of carbon to the sediments. This enrichment may result in a higher diversity of cultivable bacteria since these microorganisms, which are typically adapted to environments richer in organic matter in comparison to non-cultivable species (Fuhrman and Hagström 2008), may become dominant members of the community.

According to Schauer et al. (2010), the Walvis Ridge forms a barrier between the Cape Abyssal Plain and Angola Abyssal Plain, limiting the northward and southward flow of water at depths greater than 3000 m. Although these authors did not detect any influence of the Walvis Ridge on microbial dispersal, they reported differences between the bacterial communities of the Angola and Cape Abyssal Plains. In our cluster analysis, the two samples originating from the Angola Basin grouped separately from most of the samples collected at the Cape Basin, forming two groups (group 1 and 2) with samples 7 from the Equatorial Region and 3 from the Cape Abyssal Plain, respectively. These differences may reflect different water masses or other environmental factors that occur north and south of the Walvis Ridge. To confirm and further elucidate these differences, we need to analyze a greater number of samples from both regions.

As already mentioned, sixteen OTUs could represent new species, considering a limit of similarity of 97% in the 16S rRNA gene sequences. However, this number could be even higher if we consider a limit of 98.5 to 99%, as revised and proposed by Stackebrandt and Ebers (2006). Concerning the methodologies adopted in this study, we did not employ special conditions of cultivation, such elevated hydrostatic pressures and low temperatures, similar to that existing at the origin of the samples. Therefore, we can say that other new microorganisms can be cultured from the source of the sediment samples. We also believe that a high number of strains would allow testing hypothesis related to the distribution of the different species identified.

## Conclusions

The results of the present study demonstrated that a great part of the cultivable bacterial diversity of deep-sea environments is still unknown, and the implementation of new studies with this fraction of the microbial communities is justified. This is reinforced by the enormous biotechnological potential of these microorganisms, already demonstrated in studies by other authors (Arahal and Ventosa 2006; Simon-Colin et al. 2008; Pettit 2011). In future studies, efforts should be directed to emulate the prevailing conditions at the deep-sea floor, and to isolate a higher number of strains.

## Methods

### Sampling

**Figure 4:**
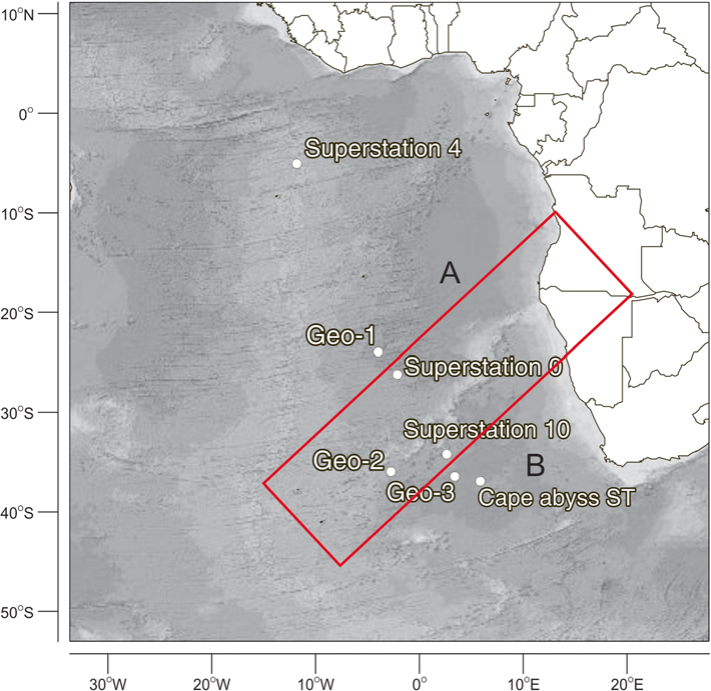
**Localization of the sampling stations.** Legend: The Walvis Ridge is highlighted in red. **A**, Angola Abyssal Plain; **B**, Cape Abyssal Plain.

The samples studied were collected during a cruise carried out between 10/25/2009 and 11/29/2009 with the vessel Akademik Loffe (Academy of Sciences – Russia). A total of eight samples of deep-sea sediments (50 g) were collected (Table 3 and Figure 4). Samples 4 and 5 were originated from the same sampling station, but from different depths in the sediment (top 10 cm layer and 70 cm below the surface). All the remaining samples were collected at the surface of the sediments. Samples were collected with trawls (samples 1, 5, 7 and 8) or push-core samplers (samples 2 and 4), aseptically transferred to sterile 15-ml Falcon tubes on board and kept at 4°C until processing, a month after collection. The in-situ temperature was not measured at the time of sampling, but Schauer et al. (2010) mentions values between 1.14 and 2.1°C in the deep-water of the Cape and Guinea Abyssal Plains.

**Table 3:** **Localization and depths of sediment samples studied**

Sample	Station	Latitude	Longitude	Depth (m)	Region	Sampler
1	Cape abyss ST	36º21'22'S	05º52'09'E	1107	CAP	Trawls
2	Geo-1	23º30'52’S	04º17'19’W	5000	AAP	Push-core
3	Geo-2	35º20’00’S	03º01’00’W	5083	CAP	Push-core
4	Geo-3, 10cm	35º50'18’S	03º26'38’E	5200	CAP	Push-core
5	Geo-3, 70cm	35º50'18’S	03º26'38’E	5200	CAP	Trawls
6	Superstation 0	25º41'70’S	02º20'72’W	4640	RT	Push-core
7	Superstation 4	04º40'22’S	12º16'20’W	1900	SEMS	Trawls
8	Superstation 10	33º40’17’S	02º35’12’E	4400	WRS	Trawls

Legend: *CAP*, Cape Abyssal Plain; *AAP*, Angola Abyssal Plain; *SEMS*, South Equatorial Region; *WRS*, Walvis Ridge Region.

### Isolation of microorganisms

For the isolation of microorganisms, samples were serially and decimally diluted from 10 ^-1^ to 10^-5^ with filtered sterile seawater as diluent. Plates of Marine Agar (MA), Marine Agar supplemented with Tween 40 (MA+Tween 40) and Marine Agar supplemented with 1% carboxymethylcellulose (MA+CMC) were inoculated with 100 μl of the different dilutions, in triplicate, and incubated for three weeks at 10°C. This temperature was chosen since it supports the growth of a wider range of bacteria (psychrophiles and cold-tolerant mesophiles). After incubation, plates were examined and colonies of different morphology were selected and transferred to new agar plates several times, for their isolation and purification. Bacterial isolates were stored in MA slants at 4°C, where they were transferred to new agar slants every four months (Bowman 2001).

### DNA extraction and 16S gene amplification

For DNA extraction, the microorganisms were cultivated in marine broth for 2 to 7 days at 15°C. The extraction was conducted with the Genomic DNA Extraction kit from Real Genomics, following the instructions of the manufacturer. The 16S rRNA gene was amplified using the primers 27F (5’- AGAGTTTGATCMTGGCTCAG-3’) and 1492R (5'TACGGYTACCTTGTTACGACTT3'). The amplification was performed in a reaction volume of 35 μl with 5 μl of 10X concentrated buffer, 1.5 mM MgCl_2_, 200 μM dNTP mix, 0.1 μM each primer, 2.5 U Taq polymerase and 20 to 40 ng template DNA. The PCR conditions were initial denaturation of 2 min at 94°C, followed by 35 cycles of 1 min at 94°C, 1.5 min at 55°C and 1 min at 72°C, and a final extension at 72°C for 3 min. The reaction product was visualized on an agarose gel (1%) under UV light after ethidium bromide staining.

### 16S gene sequencing and analysis

The amplified PCR products of bacterial gene fragments were purified and sequenced at MACROGEN sequencing company, Seoul, Korea using the automated sequencer ABI 3100 (Applied Biosystems) with BigDye Terminator Kit v. 3.1 (Applied Biosystems). Primers 27F (5'AGAGTTTGATCMTGGCTCAG3') and 1492R (5'TACGGYTACCTTGTTACGACTT3') were used for sequencing. The sequences obtained were edited with the software Vector NTI Suite 9, and compared with the NCBI database through BLAST searches. In this comparison, sequences of type strains most closely related to the sequences of the isolates were searched. For the definition of operational taxonomic units (OTUs), a similarity limit of 97% was adopted. The sequences were aligned with Muscle, and the trees were constructed from the evolutionary distances by the neighbor joining method with the software Mega (Tamura et al., 2011). The 16S rRNA gene sequences obtained in this study have been deposited in the NCBI GenBank under accession numbers JX860193 to JX860262.

### Comparison of samples according to operational taxonomic units (OTUs)

The Shannon-Wiener diversity index and evenness were calculated according to the distribution of the OTUs among the sediment samples. The samples were also compared according to the presence or absence of the different OTUs by cluster analysis. Accordingly, a dendrogram representation was generated using the Jaccard coefficient of similarity and the unweighted pair-wise grouping with mathematical averages (UPGMA) linkage method. This analysis was conducted with the Multi-Variate Statistical Package (MVSP) software (Kovach Computing Services).

## Electronic supplementary material

Additional file 1: Table S1: Identification, similarity percentage and strains in each operational taxonomic unit identified in the present study. (DOC 82 KB)
