# Supplementary material for: Phylogenetic identification of marine bacteria isolated from deep-sea sediments of the eastern South Atlantic Ocean
Source: Springerplus. 2013 Mar 22;2:127. doi: 10.1186/2193-1801-2-127 (PMC3616218; doi:10.1186/2193-1801-2-127)
Supplement: Supplementary file 1 — Additional file 1: Table S1: Identification, similarity percentage and strains in each operational taxonomic unit identified in the present study. (DOC 82 KB) [file 40064_2013_188_MOESM1_ESM.doc]

Supplementary Table. Identification, similarity percentage and strains in each operational taxonomic unit identified in the present study.

| **OTU** | **Next related type strains** | **Acession number** | **Identity** | **Strain** |
| --- | --- | --- | --- | --- |
| *Gammaproteobacteria* | |  |  |  |
| 1 | *Cobetia marina* strain DSM 4741 | NR_042065.1 | 98 - 99% | LAMA 622 & LAMA 625 |
| 2 | *Halomonas aquamarina* strain DSM 30161/*Halomonas axialensis* strain Althf1/*Halomonas meridiana* strain DSM 5425 | NR_042063.1/NR_027219.1/NR_042066.1 | 98% | LAMA 704 |
| 3 | *Halomonas boliviensis* strain LC1 | NR_029080.1 | 93% | LAMA 796 |
| 4 | *H. boliviensis* strain LC1 | NR_029080.1 | 94% | LAMA 642 |
| 5 | *H. boliviensis* strain LC1/*H. neptunia* strain Eplume1 | NR_029080.1/NR_027218.1 | 99% | LAMA 791, LAMA 809 & LAMA 879 |
| 6 | *H. boliviensis* strain LC1 | NR_029080.1 | 96% | LAMA 837 |
| 7 | *H. boliviensis* strain LC1 | NR_029080.1 | 97 - 99% | LAMA 627, LAMA 643, LAMA 644, LAMA 646, LAMA 734, LAMA 794, LAMA 797, LAMA 802, LAMA 807 & LAMA 810 |
| 8 | *H. boliviensis* strain LC1/*Halomonas neptunia* strain Eplume1 | NR_029080.1/NR_027218.1 | 95% | LAMA 645 |
| 9 | *H. sulfidaeris* strain Esulfide1 | NR_027185.1 | 94% | LAMA 634 |
| 10 | *H. sulfidaeris* strain Esulfide1 | NR_027185.1 | 96% | LAMA 638 |
| 11 | *H. sulfidaeris* strain Esulfide1 | NR_027185.1 | 98 - 99% | LAMA 632, LAMA 633, LAMA 636, LAMA 770, LAMA 786 & LAMA 838 |
| 12 | *Idiomarina loihiensis* strain L2-TR | NR_025119.1 | 93% | LAMA 617 |
| 13 | *I. loihiensis* strain L2-TR | NR_025119.1 | 96% | LAMA 683 |
| 14 | *I. loihiensis* strain L2-TR | NR_025119.1 | 99 - 100% | LAMA 691 |
| 15 | *Marinobacter excellens* strain KMM 3809 | NR_025690.1 | 99% | LAMA 842 |

Supplementary Table (continued)

| **OTU** | ***Next related type strains*** | **Acession number** | **Identity** | **Strain** |
| --- | --- | --- | --- | --- |
| 16 | *Pseudoalteromonas issachenkonii* strain KMM 3549/*Pseudoalteromonas tetraodontis* strain IAM 14160 | NR_025139.1/NR_041787.1 | 99% | LAMA 784 |
| 17 | *Psychrobacter aquaticus* strain CMS 56/*Psychrobacter vallis* strain : CMS 39 | NR_042206.1/NR_042205.1 | 96% | LAMA 624 |
| 18 | *Psychrobacter namhaensis* strain SW-242 | NR_043141.1 | 96% | LAMA 723 |
| 19 | *Psychrobacter nivimaris* strain 88/2-7 | NR_028948.1 | 98 - 99% | LAMA 639, LAMA 641, LAMA 799 & LAMA 818 |
| *Firmicutes* | |  |  |  |
| 20 | *Bacillus aerophilus* strain :28K/*B. altitudinis* strain :41KF2b/*B. stratosphericus* strain :41KF2a | NR_042339.1/NR_042337.1/NR_042336.1 | 99% | LAMA 713, LAMA 762, LAMA 781 & LAMA 892 |
| 21 | *Bacillus firmus* strain IAM 12464 | NR_025842.1 | 94% | LAMA 692 |
| 22 | *Bacillus infantis* strain SMC 4352-1 | NR_043267.1 | 99% | LAMA 732 |
| 23 | *Bacillus pocheonensis* strain Gsoil 420 | NR_041377.1 | 93% | LAMA 690 |
| 24 | *B. pocheonensis* strain Gsoil 420 | NR_041377.1 | 97% | LAMA 687 & LAMA 878 |
| 25 | *Bacillus thuringiensis* strain IAM 12077 | NR_043403.1 | 99% | LAMA 720 |
| 26 | *Brevibacillus parabrevis* strain IFO 12334 | NR_040981.1 | 99% | LAMA 739 |
| 27 | *Oceanobacillus iheyensis* HTE831 | NR_028001.1 | 93% | LAMA 706 |
| 28 | *O. iheyensis* HTE831 | NR_028001.1 | 99% | LAMA 618 & LAMA 751 |
| 29 | *Paenibacillus glucanolyticus* strain DSM 5162 | NR_040883.1 | 96% | LAMA 766 |
| 30 | *P. glucanolyticus* strain DSM 5162 | NR_040883.1 | 99% | LAMA 767 |
| 31 | *Planomicrobium okeanokoites* strain IFO 12536 | NR_025864.1 | 97% | LAMA 637 |
| 32 | *Sporosarcina saromensis* strain HG645 | NR_041359.1 | 98% | LAMA 695 |
| 33 | *Staphylococcus cohnii* subsp. *urealyticus* strain CK27 | NR_037046.1 | 96% | LAMA 616 |

Supplementary Table (continued)

| **OTU** | ***Next related type strains*** | **Acession number** | **Identity** | **Strain** |
| --- | --- | --- | --- | --- |
| 34 | *S. cohnii* subsp. *urealyticus* strain CK27 | NR_037046.1 | 97 - 99% | LAMA 817, LAMA 819, LAMA 820 & LAMA 822 |
| 35 | *Staphylococcus saprophyticus* subsp. *bovis* strain GTC 843 | NR_041324.1 | 99% | LAMA 780 |
| 36 | *Staphylococcus xylosus* strain KL 162 | NR_036907.1 | 97% | LAMA 833 |
| 37 | *Terribacillus saccharophilus* strain 002-048 | NR_041356.1 | 99% | LAMA 619 & LAMA 694 |
| *Actinobacteria* | |  |  |  |
| 38 | *Brevibacterium iodinum* strain DSM 2062 | NR_026241.1 | 97% | LAMA 701 |
| 39 | *Micrococcus luteus* strain DSM 20030 | NR_037113.1 | 98% | LAMA 702 |
| 40 | *Nesterenkonia halobia* strain DSM 20541 | NR_026197.1 | 96% | LAMA 757 |
| 41 | *Nesterenkonia halophila* strain YIM 70179/*N. halobia* strain DSM 20541 | NR_043205.1/NR_026197.1 | 97% | LAMA 754 |
